# The value of preoperative sentinel lymph node contrast-enhanced ultrasound for breast cancer: a large, multicenter trial

**DOI:** 10.1186/s12885-022-09551-y

**Published:** 2022-04-26

**Authors:** Juan Li, Hui Li, Ling Guan, Yun Lu, Weiwei Zhan, Yijie Dong, Peng Gu, Jian Liu, Wen Cheng, Ziyue Na, Lina Tang, Zhongshi Du, Lichun Yang, Saiping Hai, Chen Yang, Qingqiu Zheng, Yuhua Zhang, Shan Wang, Fang Li, Jing Fu, Man Lu

**Affiliations:** 1 Ultrasound Medical Center, Sichuan Cancer Hospital Institute, Sichuan Cancer Center, School of Medicine, No.55, Section 4, South Renmin Road, Chengdu, China; 2 Breast Surgeons Department, Sichuan Cancer Hospital Institute, Sichuan Cancer Center, School of Medicine, University of Electronic Science and Technology of China, Chengdu, China; 3 Ultrasound Medical Center, Gansu Cancer Hospital, Lanzhou, China; 4 Ultrasound Medical Center, Ruijin Hospital, Shanghai Jiao Tong University School of Medicine, Shanghai, China; 5 Ultrasound Medical Center, Affiliated Hospital of North Sichuan Medical College, Nanchong, China; 6 Ultrasound Medical Center, the Affiliated Tumor Hospital of Harbin Medical University, Harbin, China; 7 Ultrasound Medical Center, Fujian Cancer Hospital and Fujian Medical University Cancer Hospital, Fuzhou, People’s Republic of China; 8 Ultrasound Medical Center, Yunnan Cancer Hospital, Kunming, China; 9 Ultrasound Medical Center, Zhejiang Cancer Hospital, Hangzhou, 310022 China; 10 Ultrasound Medical Center, The Affiliated Cancer Hospital of Zhengzhou University, Zhengzhou, China; 11 Ultrasound Medical Center, Chongqing Cancer Hospital and Cancer Institute, Chongqing, China

**Keywords:** Breast cancer, Contrast agents, Sentinel lymph node, Ultrasonography

## Abstract

**Objective:**

The study conducted a multicenter study in China to explore the learning curve of contrast enhanced ultrasound (CEUS) for sentinel lymph nodes (SLNs), the feasibility of using this technique for the localization of SLNs and lymphatic channels (LCs) and its diagnostic performance for lymph node metastasis.

**Method:**

Nine hundred two patients with early invasive breast cancer from six tertiary class hospitals in China were enrolled between December 2016 and December 2019. Each patient received general ultrasound scanning and SLN-CEUS before surgery. The locations and sizes of LCs and SLNs were marked on the body surface based on observations from SLN-CEUS. These body surface markers were then compared with intraoperative blue staining in terms of their locations. The first 40 patients from each center were included in determining the learning curve of SLN-CEUS across sites. The remaining patients were used to investigate the diagnostic efficacy of this technique in comparison with intraoperative blue staining and pathology respectively.

**Result:**

The ultrasound doctor can master SLN-CEUS after 25 cases, and the mean operating time is 22.5 min. The sensitivity, specificity, negative predictive value, and positive predictive value of SLN-CEUS in diagnosing lymph node metastases were 86.47, 89.81, 74.90, and 94.97% respectively.

**Conclusion:**

Ultrasound doctors can master SLN-CEUS with a suitable learning curve. SLN-CEUS is a feasible and useful approach to locate SLNs and LCs before surgery and it is helpful for diagnosing LN metastases.

## Background

Breast cancer is one of the most common malignant tumors in women, accounting for 30% of all new cancer in women [1, 2]. The sentinel lymph node (SLN) is the first site of lymphatic drainage in breast cancer. It has important guiding significance for the clinical-stage, treatment, and prognostic evaluation of breast cancer patients [3]. Sentinel lymph node biopsy (SLNB) has replaced axillary lymph node dissection (ALND) as a routine surgical procedure in breast surgery [4, 5]. It can provide patients with accurate staging and reduce the incidence of surgical complications.

SLN mapping is an important step in SLNB, while tracers are believed to be the key to accurately locate the SLN and lymph channel (LC) in SLN mapping [6]. Different methods have been proposed in this context, including blue dye, radioisotopes and fluorescence [7]. The reported performance of both radioisotopes and fluorescence are better than blue dye. However, due to logistical challenges of obtaining medical grade radioisotopes and the high costs, it is only used by very few hospitals in China. The blue dye (BD) method requires surgery and may result in excessive resection of chaotically branched lymph nodes. Moreover, patients are also prone to allergic reactions, local fat necrosis, skin staining, and other adverse reactions. Thus, more alternative techniques are looking for SLNB procedure especially in China.

With the development of ultrasound technology in recent years, contrast-enhanced ultrasound (CEUS) has begun to be used to locate SLN before surgery [8–13]. In 2004, Goldberg et al. injected microbubbles around the tumor of a pig melanoma model for the first time, which confirmed that CEUS could identify draining LCs and SLNs [14–16]. Zhao et al. reported that the sensitivity and specificity of CEUS for diagnosing SLN metastasis were 100 and 52%, respectively [8]. Furthermore, Zhou et al. retrospectively compared two tracer methods (i.e., combined CEUS and blue dye vs. combined indocyanine green and blue dye) [17]. The results showed that the two methods had the same effect, and the detection rates were 98.4 and 98.1%, respectively.

Our previous work has confirmed that both two-dimensional and three-dimensional CEUS could clearly show the number and course of LC and SLN in early breast cancer [18, 19]. Moreover, two-dimensional or three-dimensional CEUS can also determine whether SLN has metastasized. Nevertheless, studies mentioned above were all conducted with small sample sizes and in single centers. In this study, we conducted a multicenter study with ten top-grade hospitals in China. The aim of this study is to investigate the learning curve of CEUS for SLNs (SLN-CEUS) and to explore the diagnostic efficacy of this technique in comparison with intraoperative blue staining and pathology.

## Methods

### Setting and participants

The study was an observatory study with a pre-defined time range, i.e. from December 2016 to December 2019. The primary endpoint of this study was to prove the feasibility of SLN-CEUS in locating SLNs and LCs in terms of the consistency rate compared with intraoperative blue staining. The secondary endpoints including 1) an evaluation of the learning curve of SLN-CEUS; 2) the number of LC and SLN detected by SLN-CEUS; 3) diagnostic performance of SLN-CUES for lymph node metastasis compared with pathology. The endpoint of this study for each enrolled patient is when the pathology report of SLNB is obtained. Ten tertiary class hospitals participated in the study, including Sichuan Provincial Cancer Hospital, Gansu Province Tumor Hospital, Ruijin Hospital, Shanghai Jiaotong University Hospital, North Sichuan Medical College Affiliated Hospital, Cancer Hospital, Hebei Province Hospital, Fujian Province Tumor Hospital, Harbin Medical University Affiliated Tumor Hospital, Cancer Hospital in Zhejiang Province, Yunnan Province Tumor Hospital, Cancer Hospital in Chongqing, and Tumor Hospital of Zhengzhou City. The ethical review board of each center approved this study.

The study inclusion criteria were: 1) age > 18 years; 2) absence of an enlarged axillary lymph node on clinical examination; 3) clinically diagnosed as carcinoma in situ or early invasive breast cancer and will undergo SLNB. The exclusion criteria were: 1) pregnancy/ lactation; 2) inflammatory breast cancer; 3) axillary lymph nodes were clinical diagnosed as positive; 4) underwent chemotherapy or radiotherapy; 5) history of breast or plastic surgery; 6) history of cardiovascular, respiratory, or immune system diseases; 7) severe allergy to ultrasound contrast agents; 8) severe blood clotting disorders. Informed consent was obtained from all enrolled patients.

The study in each center was conducted by a doctor with more than 5 years’ experience in breast ultrasound and 1 year’s experience in CEUS for other axillary mode characterization. All the participants across sites were trained with a uniform and standard procedure of operation and data collection, which was jointly developed by all sites.

We should note that all the doctors were not familiar with SLN-CEUS before the study. In order to investigate their learning curve and eliminate the impact of inter-operator’s difference in their familiarity of this technique, we considered the first 40 patients for each site as technique learning. The number of 40 was discussed and set by a group of doctors with rich experience in SLN-CEUS. The remaining patients were used to explore the diagnostic efficacy of SLN-CEUS. With the pre-defined time range of this multicenter study, four hospitals recruited less than 40 patients. Thus, we have finally six tertiary class hospitals included in this study.

**Fig. 1:**
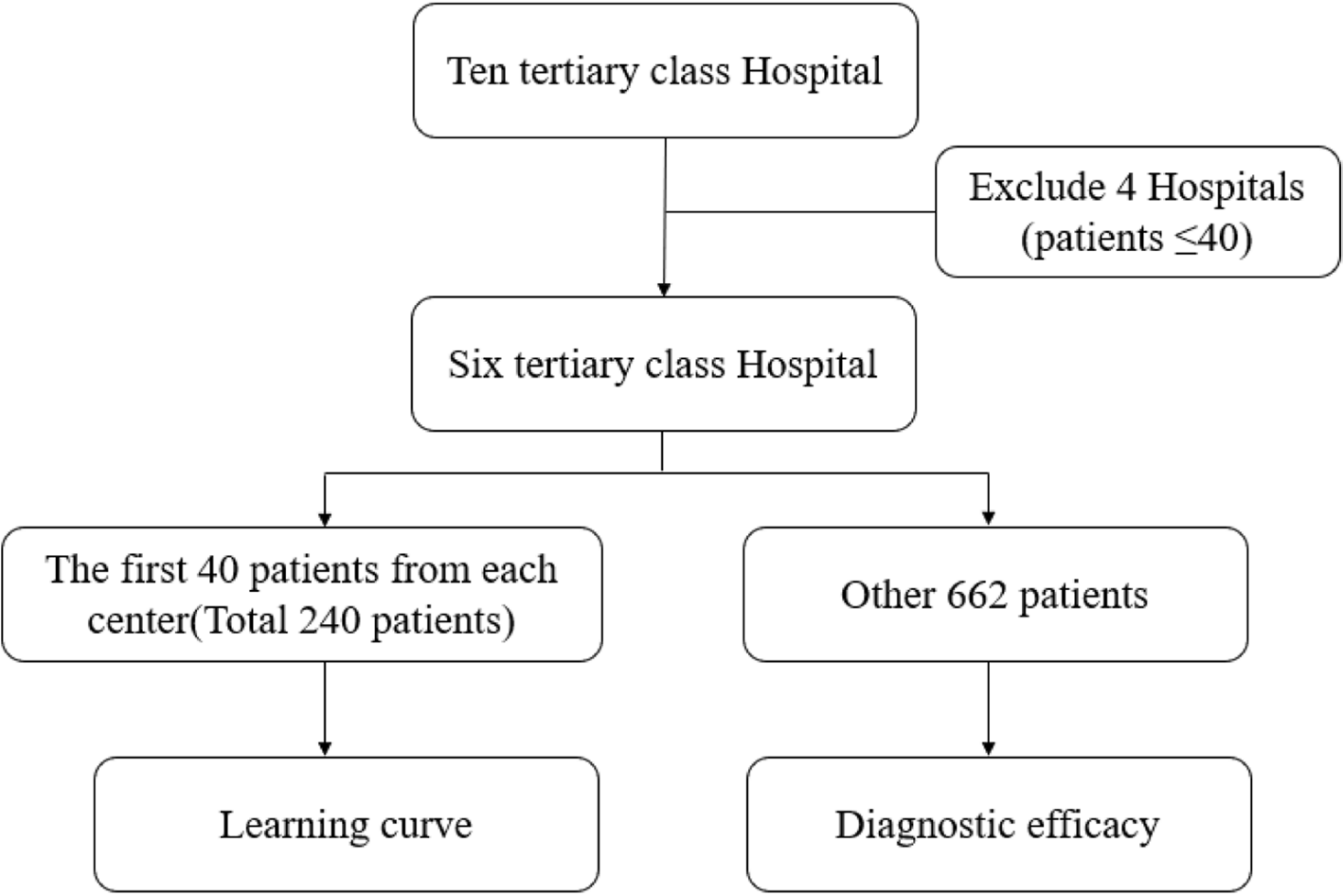
Flowcharts of the study

The flowcharts of the study showed in Fig. 1.

### SLN-CEUS examination

Different ultrasonic equipments were used for SLN-CEUS examinations in each center, including Philips iU22\Epiq7, Esaote MyLab™ Twice, Mindray Resona-7, Siemens S2000, and GE Logic E9. The instruments were uniformly calibrated prior to the start of data collection. SonoVue (Bracco spa, Milan, Italy) was used as a contrast agent and it was prepared according to reference [15, 16]. A lower mechanical index (MI) value (MI = 0.2–0.4) was used in ultrasonic equipments to reduce the damage to microbubbles.

Image depth and gain were also adjusted through a real-time dual CEUS mode. Approximately 0.6 ml of contrast agent was then injected intradermally into the periareolar area at the position of 3, 6, 9, and 12 o’clock. The SLN was defined as the first enhanced lymph node at the end of each LC. The shape and location of LCs and SLNs were identified by cross-sectional and longitudinal scans respectively. They were then marked on the body surface using a marker pen. The markers were covered by a transparent applicator for protection before SLNB. These body surface markers were then used to assess the feasibility of SLN-CEUS for the localization of SLNs and LCs in comparison with intraoperative blue staining. Operating time was defined as the total time from the beginning of ultrasound scanning to the completion of body surface markers.

### The SLNB procedure

After general anesthesia, a total of 2.4 ml of methylene blue dye was injected intradermally into periareolar area at the position of 3, 6, 9, and 12 o’clock. The surgery then began after massaging for about 5–10 min. The incision line was determined based on the body surface maker that were made from the preoperative SLN-CEUS. The surgeon might then find one or more draining blue-stained LCs. The first blue-stained lymph node was defined as the SLN. If multiple lymph nodes were identified at the end of the LC, they were all defined as the SLNs.

After dissecting the LCs along the armpit’s subcutaneous staining, their position was compared to the preoperative body surface markers. The SLN was completely excised and sent for examination. Next, the other stained lymph nodes in the axillary region were taken out for pathological biopsy. Within each center, axillary surgeries were performed by the same breast surgeon with over 5 years of surgical experience, and pathological examinations were performed by the same pathologist with over 5 years of experience performing the examinations.

### CEUS image analysis

In SLN-CEUS image analysis, the number of draining LCs, the shape and number of terminal SLNs, and the enhancement pattern of SLNs were observed. In accordance with literature report [8, 13], the examinees defined the enhancement pattern of SLN-CEUS as homogeneous enhancement, heterogeneous enhancement, or no enhancement. Homogeneous enhancement was considered as the absence of metastatic lymph nodes, while heterogeneous enhancement and no enhancement were considered as the presence of metastatic lymph nodes. The results were compared with pathological diagnosis.

### Patient characteristics

Information on patient age, body mass index (BMI) (kg/m2), tumor characteristics such as location, pathological type, as well as presence or absence of SLN identification, and the number of resected SLNs were also collected. BMI was calculated as weight in kilograms divided by height in meters squared (BMI = kg/m2).

### Statistics

The first 40 patients from each center were included in determining the overall learning curve across sites. According to an experienced radiologist, the operating time of an SLN-CEUS examination when the skill of an operator reaches a stable state was set as 25 min. CUSUM was calculated as or = Xi – XO, where Xi = operating time and XO = the time required for SLN-CEUS when the skill of an operator reaches a stable state (i.e., 25 min). The abscissa axis was the number of SLN-CEUS examinations, and the ordinate was CUSUM (or value). The curve fitting function within MATLAB software (MathWorks Inc., Natick, Mass., USA) was used to draw a polynomial function curve, and the slopes (i.e., k-value) of the curves with respect to to each examination were calculated. The learning curve analysis was done as follows: evaluate the curve fitting result by its coefficient R2; obtain the derivative function formula of the curve function; calculate the curve slope value for each SLN-CEUS examination; calculate the abscissa value when the slope value is equal to 0; calculate the curve function value using the abscissa value. The first X integer value after the curve’s peak value indicated the minimum number of SLN-CEUS examinations required for an operator to master the skill.

The remaining patients enrolled in each center were used to investigate the diagnostic efficacy of SLN-CEUS in comparison with intraoperative blue staining and pathology respectively. Intraoperative blue-stained LCs were considered as the gold standard for SLN localization. Paraffin section results were used as the gold standard for SLN metastasis diagnosis. Statistical analyses were conducted using SPSS Statistics for Windows, version 13.0 (SPSS Inc., Chicago, Ill., USA).

## Results

A total of 902 patients from six hospitals were enrolled in this study. Hospitals include Sichuan Cancer Hospital, Gansu Cancer Hospital, Ruijin Hospital of Shanghai Jiaotong University, Affiliated Hospital of North Sichuan Medical College, Hebei Cancer Hospital, and Fujian Cancer Hospital.

### The learning curve of multicenter

As mentioned, the first 40 patients in each center were enrolled to study the learning curve of SLN-CEUS. The characteristics of these patients in the six centers (numbered from 1 to 6) are presented in Tabel 1. The distribution of patients in terms of their age, lesion location, cancer stage and types are provided. The results indicated that the differences in age, lesion location, cancer stage or type of the first 40 patients between these six centers were not statistically significant (*P* > 0.05) (Table 1).

**Table 1:** Patient demographics and clinical characteristics

Variable	No. of patients (%)						
	Center 1	Center 2	Center 3	Center 4	Center5	Center 6	*P*
**Patient age**							
< 60 years	48.6	52.6	47.9	48.1	55.3	47.6	0.098
> 60 years	51.4	47.4	52.1	51.9	44.7	52.4	
Location							
**Central portion**	1.6	0.9	0.5	1.7	1.1	0.7	0.956
Upper outer quadrant	40.8	41.7	37.8	39.1	37.1	38.8	
Upper inner quadrant	17.4	18.9	20.5	20.7	19.2	20.1	
Lower outer quadrant	27.2	24.8	28.1	27.1	25.9	26.7	
Lower inner quadrant	13.0	13.7	13.5	11.4	16.7	13.7	
**Cancer stage**							
T1 (< 2 cm)	47.7	56.1	52.7	51.8	50.5	51.2	0.068
T2 (2-5cm)	52.3	43.9	47.3	48.2	49.5	48.8	
**Cancer type**							
Invasive ductal carcinoma	66.7	61.7	63.1	57.2	60.9	62.6	0.674
Invasive lobular carcinoma	22.3	24.6	27.2	27.7	26.8	26.6	
Tubular carcinoma	11.0	13.7	9.7	15.1	12.3	10.8	

The basic information of LC, SLN and SLN metastasis in six centers are shown in Table 2. The number of founded LCs in one case is in the range of [0, 3] and that of founded SLNs is in the range of [0, 4]. For the row of pathology, 0 stands for the absence of metastatic lymph nodes and 1 presents the presence of metastatic lymph nodes. The results indicated that the difference in the number of LC and the status of lymph nodes metastasis between six centers were not statistically significant among these six centers. However, the number of SLN was significantly different (*P* < 0.05), which is probably due to the very limited number of patients (i.e. 40) for learning curve study. Nevertheless, the identification rate of CEUS was not significantly different between different centers, indicating that all of the doctors participating in this study had similar skill level.

**Table 2:** Comparison of LCs and SLN in 6 hospitals (n/%)

Multicenter			1	2	3	4	5	6	*Total*	*χ*^*2*^	*P*
LC	N	0	6	1	1	2	3	4	1	9.003	0.109
		1	24	21	28	30	26	21	160		
		2	9	17	11	6	8	13	71		
		3	1	1	1	2	3	2	10		
SLN	N	0	6	0	2	1	0	0	7	14.375	0.013
		1	18	18	25	29	22	16	129		
		2	9	15	13	10	15	22	84		
		3	7	4	0	0	3	2	11		
		4	0	2	0	0	0	0	2		
Pathology		0	31	29	29	27	29	30		1.114	0.953
		1	9	11	11	12	11	10			
Detection rate			85.0%	97.5%	95.0%	92.5%	92.5%	87.5%		0.954	0.978

**Fig. 2:**
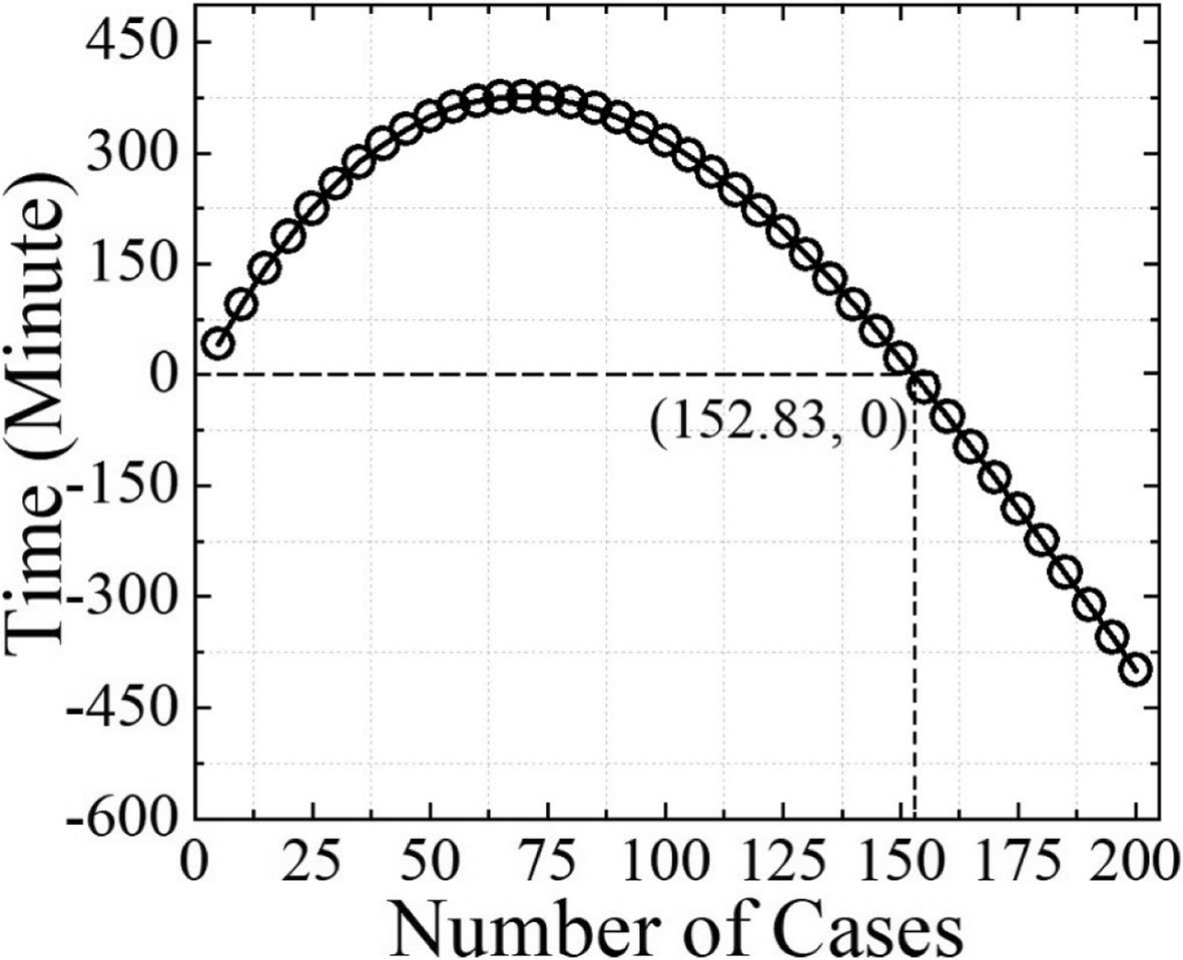
The learning curve of multicenter

The learning curve is shown in Fig. 2, where the abscissa is the number of SLN-CEUS examinations (i.e. number of cases), the ordinate is CUSUM (or value). The determination coefficient R2 of the curve function was 0.9590, which indicates that the curve fitting was done well. The peak value of the curve was located between 25 and 26, which means that the minimum number of SLN-CEUS examination required for an operator to master this technique is 25. The mean operating time of an SLN-CEUS examination is 22.5 min for each patient.

### Location of SLNs and LCs detected by SLN-CEUS

Of the remaining 662 patients, SLNs were detected in 638 of them. The detection rate was thus 96.37% (638 / 662).

To determine the cutoff level for BMI, and age that separated the patient collective with highest significance, an ROC analysis and subsequent chisquare testing were performed. Results revealed a BMI of 26 kg/m2 as the cutoff level, corresponding to a detection rate of 99.25% for patients with a BMI of up to 26 kg/m2 versus a detection rate of 90.84% for patients with a BMI of more than 26 kg/m2 (*P* = 0.004). The cutoff level calculation with regard to patient age revealed an age of 58 years as the cutoff level, corresponding to a detection rate of 98.34% for patients up to 58.4 years and 94.73% for patients above that age (*P* = 0.716).

**Fig. 3:**
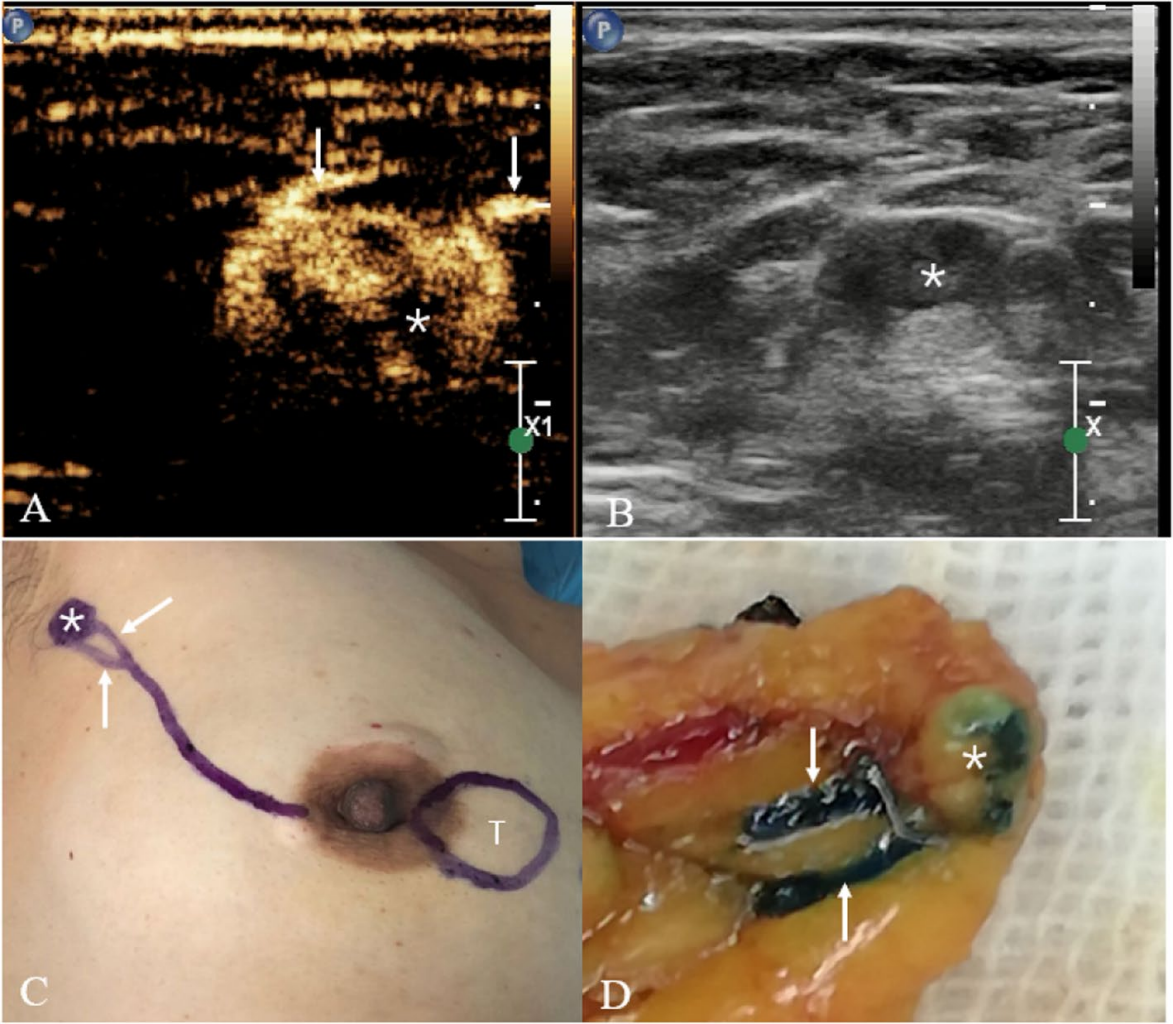
Sixty-six-year-old woman with invasive breast cancer. **A** SLN-CEUS reveals one LC (arrows) draining into one SLN (asterisk). **B** 2-D US reveals axillary lymph node. **C** Surface marks on SLN and LC made with gentian violet. **D** Comparisons of the surface marks on the LC and SLN made by CEUS during surgery; the LC contains blue dye

The mean number of detected LC in each patient is 1.25 and that of SLN is 1.42. In total, 1420 SLNs and 828 LCs were detected. The number of common LC was 1–4. The common patterns of lymphatic drainage include: 1 LC to 1 SLN, 1 LC to 2 SLNs, 2 LCs to 2 SLNs, 2 LCs to 1 SLN, and 2 LCs to 3 SLNs (Fig. 3). We used a clockwise direction to define the direction of the outflow of lymphatic drainage vessels; the area from 11:30 to 12:30 was defined as the direction of 12 o’clock, and the others were defined in a similar manner. In terms of the direction of lymphatic drainage, results showed that 43.37% of the cases were in 12 o’clock, 14.58% in 11 o’clock, 7.57% in 10 o’clock, 6.44% in 2 o’clock, 10.60% in 1 o’clock, and 16.48% in the other directions.

The locations of the detected SLN were compared with intraoperative blue staining and the consistency rate between them was 92.7%.

### SLN metastasis

Of the 1420 SLNs detected, 965 cases were presented as homogeneous enhancement in CEUS, 378 cases were with heterogeneous enhancement and 77 cases were with no enhancement. Thus, 965 cases were considered as the absence of metastatic lymph nodes, while 455 cases were considered as the presence of metastatic lymph nodes. Compared with pathology which is considered as the gold standard, the sensitivity, specificity, accuracy, positive predictive value, and negative predictive value of SLN-CEUS were 86.47, 89.81, 85.4, 74.90, and 94.97%, respectively.

## Discussion

SLN-CEUS has been increasingly used in clinical practice in recent years. The published literature shows that SLN-CEUS can clearly define SLNs in breast cancer, with a sensitivity of 80.9–100% and specificity of 70–92.3%. Nevertheless, studies reported were all conducted with small sample size in a single center. In this study, we conducted a multicenter study with ten top-grade hospitals to investigate the learning curve of SLN-CEUS.

The rising period of the learning curve corresponded to the initial stage of learning. The exploration period of the curve analysis was from the first to the 18th exercise. In this stage, the time of catheterization was long, the operators were not proficient in the operation and could not reach the operation standard, and the operation success rate was low. After accumulation, the cumulative sum value is larger, so the learning curve shows an upward trend, and the increase in range is large.

During the platform period of the learning curve, the operator’s skills gradually improved as a result of the initial exploration and learning. Compared with the rising period of the learning curve, the average surgery time was reduced, and the diagnostic accuracy was improved from the 19st to 24th exercises within the curve analysis. At this time, the observation indices gradually approached and even reached the target value. Therefore, the value decreased compared with the rising period of the curve, and the cumulative sum value after accumulation was smaller than that in the rising period. In the learning period, the curve continued to rise, but the increasing range became smaller, and the trend became slower as it entered the platform period. During this period, the slope of the curve was still positive but gradually decreased and approached zero.

During the decline period of the learning curve, the operators’ skill levels were consistently improved, and they gradually mastered the operation skills. The evaluation indices reached the preset target value, the operation speed was accelerated, and the operation success rate was higher, and the state was relatively stable. When the observation index reached the target value, the or value in the formula or = Xi - XO, was negative. Therefore, after accumulation, the cumulative sum value gradually decreased, the learning curve showed a downward trend, and the curve slope began to be negative during this period and continued to decrease. As of the 25th operation, the slope of the curve began to be negative, and the curve began to show a downward trend (i.e., entered the decline period of the learning curve). Therefore, the minimum number of operations needed to master the SLN-CEUS was 25 cases.

In this study, the SLN identification rate was 96.37%. We also found that identification rate decreased as BMI increased. The underlying reason for this observation may be due to changes in the distribution and density of lymphatic vessels draining the breast when fat replacement occurs. The consistency rate between SLN-CEUS and BD in terms of identified SLNs location was 92.7%. We should note that the location of SLNs identified by SLN-CEUS was compared with findings from BD as BD is currently the most widely used method in China. Although the reported performance of BD is inferior to radioisotopes and fluorescence, this comparison could be most beneficial for those regions or countries that have limited access to radioisotopes or fluorescence.

The sensitivity, specificity, positive predictive value, and negative predictive value of SLN were 86.47, 89.81, 74.90, and 94.97%, respectively. The sensitivity and specificity were lower than that of our previous study (86.47% vs. 96.82 and 89.81% vs. 91.91%, respectively) [19]. However, the positive and negative predictive values were higher than those of Zhao et al. (74.90% vs. 64.9 and 94.97% vs. 43.4%) [8]. This may be due to the different experience-levels of each center. In this study, some SLNs without metastasis showed a heterogeneous enhancement pattern. Possible explanations include an insufficient contrast medium, that the lymphangiosis was too thin, or that the inflammatory reaction caused by a lymph node biopsy in 1 week leads to the uneven enhancement of the SLN during CEUS. Repeated injections may solve this problem. At the same time, we also found that in some patients with metastatic SLNs, CEUS showed homogeneous enhancement. In some of these patients, immunohistochemistry showed that the isolated tumor cell clusters were less than 2 mm in diameter (i.e., micrometastasis). Our future work will further strengthen the diagnosis of micrometastasis combined with molecular imaging.

There were some limitations in this study. We only assessed the current status of SLNs. First, it would be more meaningful with a long-term follow-up to observe the survival rate and recurrence rate of these patients. Second, ultrasound equipments used in different centers were not from the same manufacturer. It remains to be explored and no one has ever investigated whether there are significant differences between different manufacturers in terms of the performance of SLN-CEUS. Third, it could be interesting if we combine SLN-CEUS with BD and compare this combined usage with BD only. This will require a case-control study with bigger sample size. Last but not least, the result of this study may have limited benefits for those regions or countries that have good access to radioisotopes or fluorescence. Since the reported performance of radioisotopes, fluorescence or the combination of BD and isotopes are superior to BD, these methods will be taken into account to further evaluate SLN-CEUS when conditions permit in the future.

## Conclusion

In conclusion, SLN-CEUS can clearly show the direction and path of LC and has value in the localization of LC and SLNs. SLN-CEUS has a learning curve. The ultrasound doctor can master this technique after 25 cases, and the mean operating time is 22.5 min. The sensitivity, specificity, negative predictive value, and positive predictive value of SLN-CEUS in diagnosing lymph node metastasis were 86.47, 89.81, 74.90, and 94.97% respectively.

## Data Availability

The datasets used and analysed during the current study available from the corresponding author on reasonable request.

## Abbreviations

SLN: Sentinel lymph node
CEUS: contrast-enhanced ultrasound
LC: Lymph channel
SLNB: Sentinel lymph node biopsy
ICG: Indocyanine green fluorescence
LN: Lymph node
ALN: Axillary lymph node
